# Highlights of the society for immunotherapy of cancer (SITC) 27th annual meeting

**DOI:** 10.1186/2051-1426-1-4

**Published:** 2013-05-29

**Authors:** David F Stroncek, Cornelis JM Melief, Luciano Castiello, Alessandra Cesano, Martin A Cheever, Sara Civini, Begonya Comin-Anduix, Thomas F Gajewski, Philip D Greenberg, Pawel Kalinski, Howard L Kaufman, Michael H Kershaw, Samir N Khleif, Francesco Marincola, William Merritt, David H Munn, Daniel J Powell, Nicholas P Restifo, Steven A Rosenberg, Raj K Puri, Howard Streicher, Aladar A Szalay, Cassian Yee, Laurence Zitvogel, Antoni Ribas

**Affiliations:** 1 Cell Processing Section, Department of Transfusion Medicine, Clinical Center, NIH, Bethesda, MD, USA; 2 Department of Immunohematology and Blood Transfusion, Leiden University Medical Center, Leiden, Netherlands; 3 Nodality, Inc, South San Francisco, CA, USA; 4 Fred Hutchinson Cancer Research Center and Cancer Immunotherapy Trials Network (CITN), Seattle, WA, USA; 5 Jonsson Comprehensive Cancer Center, University of California Los Angeles, Los Angeles, CA, USA; 6 Department of Medicine, Section of Hematology/Oncology, The University of Chicago, Chicago, IL, USA; 7 Department of Immunology, School of Medicine, University of Washington, Seattle, WA, USA; 8 Department of Surgery, University of Pittsburgh Cancer Institute, Pittsburgh, PA, USA; 9 Department of Immunology, University of Pittsburgh, Pittsburgh, PA, USA; 10 Department of Bioengineering, University of Pittsburgh, Pittsburgh, PA, USA; 11 Rush University Cancer Center, Rush University Medical Center, Chicago, IL, USA; 12 Cancer Immunology Research Program, Peter MacCallum Cancer Centre, Melbourne, Australia; 13 Georgia Health Sciences University, Augusta, GA, USA; 14 Sidra Medical and Research, Centre, Doha, Qatar; 15 National Cancer Institute, Bethesda, MA, USA; 16 University of Pennsylvania, Philadelphia, PA, USA; 17 Surgery Branch, National Cancer Institute, NIH, Bethesda, MD, USA; 18 US Food and Drug Administration, Center for Biologics Evaluation and Research, Bethesda, MD, USA; 19 Genelux Corporation, San Diego, CA, USA; 20 MD Anderson Cancer Center, Houston, TX, USA; 21 Institute Gustave Roussy, Villejuif, France

**Keywords:** Immunotherapy, Cancer, Adoptive cellular therapy

## Abstract

The 27^th^ annual meeting of the Society for Immunotherapy of Cancer (SITC) was held on October 26–28, 2012 in North Bethesda, Maryland and the highlights of the meeting are summarized. The topics covered at this meeting included advances in cancer treatment using adoptive cell therapy (ACT), oncolytic viruses, dendritic cells (DCs), immune check point modulators and combination therapies. Advances in immune editing of cancer, immune modulation by cancer and the tumor microenvironment were also discussed as were advances in single cell analysis and the manufacture and potency testing of tumor infiltrating lymphocytes (TIL).

## Immunology and cancer

The immune system plays an important role in cancer progression and cancer therapy. In many ways the immune system of cancer patients is suppressed and the tumors actively evade immune surveillance. T cells, especially those within tumors, are impaired functionally and tumors induce T and NK cell apoptosis. The tumors also create a unique microenvironment that promotes tumor growth and blocks the anti-tumor activities of T cells.

In a keynote address, Robert Schreiber (Washington University, Saint Louis, MI) described that cancer cells go through 3 phases of immune editing; elimination, equilibrium and escape [1]. In the elimination phase newly emerging cancer cells are recognized and eliminated. In the equilibrium phase the cancer cells are recognized but can no longer be eliminated and in the escape phase the tumor cells are no longer recognized. Immune editing is dependent on CD8+ cells, CD4+ cells and the recognition of target antigens. Mutated antigens are often involved with immune editing of tumor cells. The expression of mutated antigens allows tumors to be recognized by the immune system. The loss of these mutated antigens allows the tumors to escape the immune system.

As described by Theresa Whiteside (University of Pittsburgh, Pittsburg, PA) during the Richard Smalley Memorial Award Lecture, tumors induce many changes in host immune response that contribute to immune escape. These changes include the induction of T cell apoptosis and expansion of T_REG_ cells. Many cancers induce reductions in the quantities of circulating T cells which likely influences clinical outcomes. The reduction in the number of T cells and proliferation of T_REG_ cells is mediated in part by tumor-derived exosomes (TEX) whose levels are increased in the sera of cancer patients [2]. These exosomes express a number of cell surface receptors and ligands including FAS ligand and exosomes isolated from the sera of cancer patients have been shown to induce FAS mediated apoptosis of T cells. Tumor derived exosomes also promote T_REG_ cell proliferation. They also interact with monocytes to yield myeloid-derived suppressive cells (MDSC).

As pointed out by David H. Munn (Georgia Health Sciences University, Augusta, GA), the indoleamine 2,3-dioxygenase (IDO) pathway is also likely a major contributor to tumor associated immune suppression. IDO is a natural factor that is a counter immune regulator in that it is induced by inflammation but it is immunosuppressive [3]. It regulates both the innate and adaptive immune responses. IDO is expressed by a wide range of cancer cells and increased levels are associated with poor clinical outcomes. IDO is also expressed by dendritic cells (DCs) in tumor draining lymph nodes and FOXO3 induces IDO expression in DCs [4].

The down-regulation of NF-κβ activity may also contribute to immune suppression in tumor-bearing hosts. NF-κβ activity is down-regulated in T cells from hosts with tumors. The reduced NF-κβ activation results in reduced T cell survival, reduced Th1 and Th17 differentiation and increased T_REG_ cell differentiation. Cesar Evaristo and colleagues (University of Chicago, Chicago, IL, USA) have found that the forced overexpression of NF-κβ in mice resulted in fewer T_REG_ cells and increased frequency of interferon-γ producing T cells and enhanced rejection of B16 melanoma cells that had been engineered to express the model SIYRYYGL (SIY) antigen [5].

Patients with chronic hepatitis C virus (HCV) infection often develop hepatocellular carcinoma and recent data suggests that the NK cell function is abnormal in patients with chronic HCV infection. Maria Libera Ascierto and colleagues (Clinical Center, NIH, Bethesda, MD, USA) have been evaluating the role of NK cells in the outcome of patients with HCV infections. The transcriptome of peripheral blood NK cells from chronically viremic treatment-naïve HCV patients was compared with patients who spontaneously achieved virus eradication and healthy subjects. NK cells from patients who spontaneously eradicated HCV upregulated genes involved with activation of NK cell function and cells from chronically viremic patients had enhanced expression of genes involved with interferon signaling and IL-15 production.

## Tumor microenvironment

The tumor microenvironment is increasingly been identified as having a critical role in tumor cell growth and rejection. Michael Kershaw and colleagues (Peter MacCallum Cancer Center, Melbourne, Australia) have found differences in microenvironment due to tumor location which affect response to therapy [6]. Immunotherapy consisting of antibodies directed to death receptor 5 (DR5), CD40 and CD137 (4-1BB) works less well in orthotopically implanted renal cell tumors than subcutaneously implanted tumors. The number of immune cells in the renal and subcutaneous tumor microenvironments was similar, but their characteristics differed. More M2 macrophage and Th2 cell genes were expressed by cells in the microenvironment of kidney tumors than in subcutaneous tumors. The vasculature also differed among the tumors as more blood vessels were present in kidney tumors.

While some critical aspects of the microenvironment have yet to be completely understood, it is clear that the presence of lymphocytes is an important factor. Lymph node-like structures, identified in melanoma microenvironment, are associated with improved responses to immunotherapy. James Mule and colleagues (Moffitt Cancer Center, Tampa, FL, USA) studied a 12-chemokine gene expression signature on arrays of 14,492 solid tumors across 24 different tissue types [7]. The twelve genes were CCL2, CCL3, CCL4, CCL5, CCL8, CCL18, CCL19, CCL21, CXCL6, CXCL10, CXCL11 and CXCL13. Among melanoma samples, those with a signature demonstrating up-regulation of all 12 genes exhibited unique lymph node-like structures. The expression of these 12 genes was also found to be associated with better overall survival. The expression of these 12-cytokine genes may be useful in the selection of patients most suitable for immunotherapy.

## Targeting immune suppression

### Antibodies to PD-1 and PD-L1

A mechanism of immune escape involves the PD-1 and PD-L1 pathway. PD-1 is up-regulated by T cells chronically activated by antigen stimulation, and its engagement by PD-L1 on tumor cells down-regulates the ability of activated T cells to produce cytokines, proliferate and kill target cells. PD-L1 is expressed by many tumors. Antibody blockage of the PD-1/PD-L1 pathway can induce tumor regression in several tumor models. Recent studies by Xiufen Chen and Justin Kline (University of Chicago, Chicago, IL, USA) suggest that blockade of the PD-1/PD-L1 pathway may not only result in the re-activation of effector T cells, but may also prevent the induction of T_REG_ cells.

The PD-1/PD-L1 pathway appears to play a key role in immune suppression in multiple myeloma. Myeloma cells express high levels of PD-L1 and myeloma-infiltrating T cells express increased levels of PD-1. Tyce Kearl, Weiqing Bing and Bryon Johnson (Medical College of Wisconsin, Milwaukee, W, USA) used a murine model of multiple myeloma to show that anti-PDL1 can enhance the clearance of myeloma by tumor-experienced T cells when it was combined with lymphodepleting irradiation. Both host CD4^+^ and CD8^+^ cells were required to improve killing of myeloma cells. Interestingly, anti-PD-L1 and lymphodepletion did not improve survival in two other solid tumor models.

The potential of PD-1/PD-L1 blockade has led to the clinical development of antibodies capable of blocking this pathway. One of the potential limitations of anti-PD-L1 therapy is that the antibody may reduce lymphocyte life span by binding to PD-L1 expressed by circulating T cells. The cells are cleared by an Fc dependent mechanism, antibody-dependent cell-mediated cytotoxicity (ADCC). Bryan Irving (Genentech, South San Francisco, CA, USA) reported the results of testing an anti-PD-L1 with Fc domain that has been altered to reduce ADCC clearance and lymphopenia. Preclinical studies using tumor bearing mice have found that the modified anti-PD-L1 was effective as a single agent and in combination with tumor-targeted therapies.

Another recently developed antibody capable of blocking this pathway is MEDI4736 which is also specific for PD-L1. Ross Stewart and collaborators (MedImmune, Cambridge, UK and Astra Zeneca Waltham, MA) reported that this antibody only activates T cells in the context of an activated T cell receptor (TCR) signal. This antibody has been shown to inhibit tumor growth in animal models.

### Anti-CTLA-4

Antibody blockade of the CTLA-4 pathway is another mechanism that is useful in overcoming cancer-induced T cell suppression. Anti-CTLA-4 has been found to induce durable remissions in some patients with melanoma. Luana Calabro and collaborators (University Hospital of Siena, Siena, Italy and MedImmune, Gaithersburg, MD, USA) reported that one such antibody, tremelimumab, has beneficial effects when used in combination with platinum-based chemotherapy to treat malignant mesothelioma. Among the 29 patients treated in a phase II clinical trial, two patients had partial clinical responses and four had prolonged stable disease and one patient remained alive 28 months after therapy.

It appears that one of the mechanisms by which anti-CLTA-4 enhances T cell responses to tumors is by epitope spreading. Pia Kvistborg and colleagues (Netherlands Cancer Institute, Amsterdam, Netherlands) used a high-throughput methodology to study cytotoxic T cell epitopes before and after the administration of the anti-CTLA-4 antibody ipilimumab, in 26 patients with melanoma. They found significant increases in the number of melanoma-associated epitopes recognized by CD8+ T cells after ipilimumab but the overall magnitude of melanoma-specific T cell responses was not changed.

### MicroRNA

Some investigators are studying microRNA (miR) as a method to reverse cancer-associated immune suppression. Amy Heimberger and colleagues (University of Texas, MD Anderson Cancer Center, Houston, TX, USA) have found that the expression of miR-124, a modulator of the T cell transcription factor Signal Transducer and Activator Transcription 3 (STAT3), is down-regulated in gliomas. The up-regulation of miR-124 in glioma cells increased T cell proliferation and inhibition of FOXP3^+^ T_REG_ cells. The administration of miR-124 in murine models of glioma resulted in anti-glioma effects suggesting that miR-124 may be a novel immune-activating agent for glioma therapy.

## IL-15 therapy

IL-15 has recently become available for use in early phase clinical trials as an alternative to IL-2. IL-15 is a member of the γ-chain family of cytokines and it increases the proliferation, survival and function of T and NK cells [8]. Alternative forms of IL-15 are being developed and tested. When natural IL-15 is released *in vivo* it is not stable, but circulating IL-15 exists naturally in association with the IL-15 receptor alpha (IL-15Rα) to form a stable IL-15/IL-15Rα heterodimer [9]. Further studies by Cristina Bergamaschi and colleagues (National Laboratory for Cancer Research, Frederick, MD, USA) have compared the pharmacokinetics of single chain IL-15 and IL-15/IL-15Rα heterodimer in mice and rhesus macaques and found that, following intravenous administration, the half-life of IL-15/IL-15Rα is about 6 times longer than IL-15 single chain. The intravascular levels of the heterodimer are higher and more stable when it is given subcutaneously than intravenously; when the heterodimer is given as a subcutaneous injection the plasma levels of IL-15 persist for 72 hours. In rhesus macaque, five subcutaneous injections of the heterodimer repeated every 3 days resulted in significant expansion of γδ, CD8+ T and NK cells in the peripheral blood.

## Adoptive cell transfer (ACT) therapy

The clinical application of ACT continues to grow and clinical response rates continue to improve [10]. As pointed out by Laszlo Radvanyi (MD Anderson Cancer Center, Houston, TX, USA) the use of the classic tumor infiltrating lymphocyte (TIL) immunotherapy is growing and many new TIL-based regimens are being developed. Nine centers are currently using TIL to treat patients with metastatic melanoma and it is estimated that over 300 patients have been treated. In these independent trials, TIL therapy has reproducibly been shown to result in objective clinical responses with response rates reported in up to 70% of treated melanoma patients [11]. The administration of non-myeloablative leukoreductive therapy prior to TIL infusion has improved clinical outcomes by increasing the availability of the serum cytokine IL-7 and IL-15 levels, opening T cell niches and eliminating T_REG_ cells and MDSC. A variety of leukoreduction protocols have been used including cyclophosphamide alone, cyclophosphamide plus fludarabine and cyclophosphamide/ fludarabine/total body irradiation (TBI) [11,12]. For instance, Cassian Yee (MD Anderson Cancer Center, Houston, TX, USA) reported that conditioning patients with high dose cyclophosphamide alone followed by the infusion of peripheral blood mononuclear cell-derived, antigen specific CD8+ CTL clones in melanoma patients has resulted in the long-term persistence of T cells and, that differentiated effector T cells could revert to a central memory phenotype in vivo following adoptive transfer [12].

While TIL therapy was already known to be a promising therapy, several road blocks had hindered its broader use and commercialization. Robert Keefe (Lonza, Walkersville, MD, USA) pointed out that, from a cell manufacturer’s view, the traditional manufacturing protocols for TIL production were: 1) long, requiring 5 to 7 weeks to complete, 2) labor intensive, 3) used large quantities of reagents and supplies and 4) required peripheral blood leukocytes (PBL) cells for the rapid expansion process. Additionally, TIL potency biomarkers were not yet identified. However, as highlighted by multiple speakers, substantial, progress has been made in all of these areas and has thus garnered increased commercial interest.

The long duration of TIL production is due, in part, to the practice of selecting tumor-reactive TIL for rapid expansion. Several approaches can shorten TIL production. One approach has been to forgo the selection of tumor reactive T cells entirely and begin TIL rapid expansion immediately after they are isolated. The cells produced by this method are known as “young TIL” [13]. Another approach involves the selection of tumor-specific T cells expressing activation markers. Alena Gros and colleagues (Surgery Branch, NCI, Bethesda, MD, USA) have found that among fresh TIL isolated from melanoma tumor digests, the subpopulation of tumor-specific T cells isolated with MART-1 peptide-MHC tetramers expressed higher levels of three negative co-stimulatory molecules that are expressed by chronically stimulated T cells: PD-1, LAG-3 and TIM-3 and a positive co-stimulatory molecule, 4-1BB. They also isolated and expanded fresh TIL subsets and found that tumor reactivity was preferential in effector-derived cells expressing PD-1, LAG-3, TIM-3 and 4-1BB. This suggests that these markers could be used to enrich TIL for melanoma-reactive T cells.

TIL rapid expansion has been further improved by changing the type of flasks used for TIL expansion. At some centers, TIL are now being expanded in gas-permeable flasks rather than traditional T-flasks and bags. TIL can be grown at a greater density in gas-permeable flasks which results in the use of less media [14]. TIL rapid expansion in gas-permeable flasks rather than T-flask and bags requires approximately 5- to 8-fold less media and media supplements including cytokines and AB serum. This results in a significant reduction in the cost of TIL production.

An additional difficulty with the typical methods used to expand TIL is that irradiated pooled allogeneic peripheral blood leukocytes (PBL) collected from healthy subjects are used as feeder cells to stimulate TIL rapid expansion. It would be desirable to have an alternate off-the-shelf, widely available product for TIL expansion. Artificial antigen presenting cells are being evaluated as a substitute for allogeneic PBL feeder cells for TIL rapid expansion. Paramagnetic beads coated with anti-CD3 and anti-CD28 can be used to stimulate the rapid, but low level expansion of TIL, however, these beads also result in a preferential increase in CD4+ cells in the final product. Daniel Powell and colleagues (University of Pennsylvania, Philadelphia, PA, USA) have been testing off-the-shelf cell artificial antigen presenting cells (APCs) as feeder cells for the rapid expansion of TIL [15]. They are using K562 cells that are genetically engineered to express Fc receptors and costimulatory ligands, such as 4-1BBL. The engineered artificial APCs are then loaded with anti-CD3/anti-CD28 antibodies allowing them to function as APCs. These artificial APCs have been used to expand TIL from melanoma and ovarian cancer. The degree of expansion and cell characteristic were similar to those expanded with allogeneic PBL but at lower TIL:APC ratios, meaning fewer feeder cells are required. In addition, these artificial APCs maintained a favorable CD8/CD4 ratio and FOXP3+ CD4+ cell frequency. Artificial APCs have the capacity to expand both “young” TIL and TIL selected from tumor-reactive cytotoxic T cells. Compared to allogeneic feeder cells, artificial APCs represent a more standardized “off-the-shelf” cellular platform for TIL expansion.

As discussed by multiple presenters during the meeting, several TIL characteristics that are associated with better clinical outcomes and likely a more potent product have now been identified. The administration of TIL products with longer telomeres, increased expression of the co-stimulator molecule CD27 and expression of CD45RO^+^ and CD28^bright^ are associated with better clinical outcomes [11]. New work is aimed at identifying the functional attributes of persisting TIL [16]. The infusion of greater number of TIL and longer intravascular persistence have also been associated with improved clinical outcome [17]. Faster reconstitution of CD4+, Foxp3+ cells after TIL therapy has been correlated with worse clinical outcomes [18]. In addition, TIL products with a more differentiated effector phenotype of the CD8+ population and a higher frequency of CD8+ T cells co-expressing the negative costimulation molecule B- and T-lymphocyte attenuator (BTLA) are associated with better clinical outcomes [17].

Since results emerging from TIL therapy trials suggest that clinical outcome is dependent in part on the number of TIL infused, efforts are being made to increase the quantity of TIL produced from each patient. Yong Qing Li and Cassian Yee (Fred Hutchinson Cancer Research Center Seattle, WA, USA and MD Anderson Cancer Center, Houston, TX, USA) have found that the expansion of CTLs in culture can be increased by the depletion of T_REG_ cells with anti-CD25 and treatment with IL-21. Furthermore, exposure to IL-21 during in vitro priming led to the generation of helper-independent CTL with a central memory phenotype (CD45RO+, CD28^hi^, CD62L^hi+^, IL-2+), a strategy which Yee has now implemented in first-in-man clinical studies [19,20]. Interestingly, as pointed out by Pamela S. Ohashi (Ontario Cancer Institute/University Health Network, Toronto, Canada), the presence of NK cells in TIL have been associated with reduced proliferation and altered cytokine production of CD8+ T cells. In addition, NK cells have an immune regulatory function [21]; they have been found to limit CD8+ T mediated toxicity, rationalizing their depletion from TIL products for infusion. Together, findings such as these highlight the great advancements being made in immunology and technology which makes it more feasible than ever to efficiently generate autologous tumor-reactive TIL products of high quality and quantity for use in adoptive immunotherapy of cancer.

## Genetically engineered T cells

In order to improve ACT therapy, T cells are being genetically engineered in a number of ways. Investigators lead by Steven A. Rosenberg (Surgery Branch, NCI, Bethesda, MD, USA) have engineered TIL to express IL-12 in order to improve their effectiveness [22]. Conferring the ability of tumor antigen-specific T cells to produce IL-12 greatly improves their function in a variety of pre-clinical studies [23,24]. Since IL-12 can cause systemic toxicity, they have been attempting to focus IL-12 production and prevent systemic toxicity by including a nuclear factor of activated T cells (NFAT) transcription factor in addition to IL-12 in the vector used to engineer TIL. This results in T cells that transcribe IL-12 only when the TCR is triggered. Autologous T cells are also being engineered to express high affinity TCRs. One of the challenges of this approach is to target antigens that have enough specificity to the tumor so that cancer regresses without causing off target toxicity and complications. Some clinical trials have used T cells engineered to express high affinity TCRs that recognize MART-1 and NY-ESO [25,26].

The preliminary results of early phase clinical trials of T cells that have been engineered to express chimeric antigen receptors (CAR) have been very promising and research in this area is growing rapidly. Three centers have reported the results of anti-CD19 CAR T cell clinical trials treating B cell lymphomas and leukemias and while the result at all three centers have been promising, there were several differences among the trials [27-29]. The vectors used in the trials differed: two different scFv portions of anti-CD19 were used, two different co-stimulatory molecules, CD28 and 4-1BB, were used and both retro and lentiviral vectors were used. Leukocyte depletion is important for the success of this therapy [29] and the pre-treatment leukoreduction protocols also differed among the three centers.

While the results of anti-CD19 CAR T cell therapy have been encouraging, the therapy itself has been associated with some toxicity including cytokine storm, tumor lysis syndrome and depletion of CD19+ B cells [27,28]. As a result, Michel Sadelain and colleagues (Memorial Sloan-Kettering Cancer Center, New York, NY, USA) are attempting to improve the safety of CAR T cell therapy. To limit off target toxicity, tumor sensing T cell are being developed [30,31]. One approach is to engineer T cells so that they recognize two antigens, thereby being less likely to cause off target cytotoxicity. Another approach to limit toxicity is to use transiently expressed RNA vectors rather than permanently expressed lentiviral or retroviral vectors. Using murine models, Nathan Singh and colleagues (University of Pennsylvania and Children’s Hospital of Philadelphia, Philadelphia, PA, USA) have found that multiple treatments with RNA CD19 CAR T cells were as effective in treating acute lymphocytic leukemia (ALL) as one treatment with lentivirus CD19 CAR T and multiple RNA GD2 CAR T cell doses were nearly as effective in treating neuroblastoma as a single dose of lentivirus GD2 CAR T cells.

## Improving ACT therapy by using less differentiated T cells

Several ACT clinical trials have found that prolonged persistence of adoptively transferred CD8^+^ T cells is associated with better clinical outcomes. Nicholas Restifo (Surgery Branch, NCI, Bethesda, MD, USA) reported that investigators are attempting to use CD8^+^ T cells with a younger or more stem-like phenotype which have a longer *in vivo* life-span and greater proliferative potential for clinical therapy [32]. Preclinical studies have shown that naïve (T_N_), stem memory (T_SCM_) and central memory (T_CM_) CD8^+^ T cells have better engraftment and anti-tumor efficacy than more differentiated effector memory (T_EM_) and effector (T_EFF_) CD8+ T cells [33]. Christopher Klebanoff and colleagues (Surgery Branch, NCI, Bethesda, MD, USA) have found that memory (T_MEM_) CD8+T cells can induce the differentiation of T_N_. They found that when T_MEM_ were present, activated T_N_ differentiated more rapidly than activated T_N_ alone [34]. This precocious differentiation was TCR-ligation dependent and required cell-to-cell contact. Further studies found that it was blocked by disruption of FasL-Fas signaling and was reproduced by exogenous FasL trimers in absence of T_MEM_ cells. These studies suggest that the removal of more differentiated T_EM_ and T_EFF_ cells from adoptive cellular therapies is necessary to prevent the corruption of the full potential of less differentiated anti-tumor T cells.

Stephen Forman and colleagues (City of Hope National Medical Center, Duarte, CA, USA) are attempting to improve CAR anti-CD19 therapy by using as starting material peripheral blood T_CM_ rather than a mixed peripheral blood T cell population. T_CM_ have a unique capacity for self-renewal, proliferation, persistence and an ability to differentiate into effector T cells. The CD8^+^CD45RO^+^CD62L^+^T_CM_ cells were obtained by depleting peripheral blood leukocytes of those cells expressing CD4, CD14 and CD45RA and then selecting cells expressing CD8 and CD62L. The isolated cells are then stimulated with anti-CD3/ anti-CD28 beads, transduced with a vector containing an anti-CD19 CAR and expanded with IL-2 and IL-15 for 3 to 6 weeks. Under a phase I/II clinical trial these autologous cells are being given to patients with non-Hodgkin lymphoma two days after receiving an autologous hematopoietic stem cell transplant.

Philip D. Greenberg and colleagues (University of Washington, Seattle, WA, USA) are using a novel combination of cytokines to maintain a young phenotype during T cell culture and expansion. The cytokine IL-21 arrests T cell differentiation and IL-15 promotes T_CM_ cell differentiation [35,36]. Expansion of T cells with IL-21, IL-7 and IL-15 produces cells that retain a central-memory-like phenotype (CD62L^+^, CCR7^+^, CD28^+^) [20,37].

Another approach to obtaining less differentiated cells for adoptive cell transfer appears to be blockage of T cell glycolysis. Madhusudhanan Sukumar and colleagues (Surgery Branch, NCI, Bethesda, MD, USA) have found that cellular metabolism influences T cell fate and function. They found that T_N_ cells which relay on fatty acid oxidation as a primary source for ATP generation, shifted to a glucose metabolism following antigen stimulation and effector differentiation. Blockade of glycolysis during T cell priming by 2-deoxyglucose (2-DG) prevented differentiation and the treatment of tumor-bearing mice with 2-DG treated T cells resulted in increased tumor-infiltration, cytokine release and tumor regression than control cells.

## Dendritic cell therapy

DC therapy is showing promising results in several different types of cancer. Brian Czerniecki and colleagues (University of Pennsylvania, Philadelphia, PA, USA) have given DC vaccines to patients with HER-2/neu overexpressing ductal carcinoma in situ (DCIS) prior to surgical resection [38]. The DCs were pulsed with six MHC class II peptides and two HLA-A2.1 binding class I binding peptides and were given at four weekly intervals. Post immunization T cell responses were documented using ELISPOT in 85% of the 27 patients against MHC class I peptides and in 88% to MHC class II peptides. The vaccine appeared to have been of some clinical benefit in five patients that displayed no evidence of residual DCIS and HER-2 expression was undetectable in 11 patients. The post vaccine surgical specimens showed increased infiltration of CD4+ T cells, CD8+ T cells and CD20+ B cells.

DC vaccines have also been used to treat patients with metastatic melanoma and the availability of an autologous melanoma cell line. Robert Dillman and colleagues (Hoag Institute for Research and Education, Hoag Hospital, Newport Beach, CA, USA) treated 42 patients either with irradiated autologous proliferating tumor cells or DCs pulsed with these tumor cells. The tumor cells or DCs were given once weekly for 3 weeks and then monthly for 5 months. The survival was significantly better in patients given the DCs with a 2-year survival of 72% compared to 31%.

Sipuleucel-T is an autologous DC-like immunotherapy approved for the treatment of castrate resistant prostate cancer. Recent studies have shown that this therapy affects the immune cell infiltrate in the prostate cancer microenvironment. In a phase II clinical trial sipulecel-T has been given to 42 patients prior to radical prostatectomy and Lawrence Fong and colleagues (Helen Diller Family Comprehensive Cancer Center, USCF, San Francisco, CA, USA) have investigated the prostate biopsies of these patients. They found that sipuleucel-T increased infiltration of T and B cells in the microenvironment. They also documented systemic T and B cell activation.

Approximately 20% of all cancers are associated with viral infections. The oncogenic virus human papilloma virus (HPV) causes cervical cancer, vulvar cancer, anal cancer and head and neck cancer. Cornelis Melief and colleagues (Leiden University Medical Center, Leiden, Netherlands) have developed a long peptide vaccine to treat HPV associated cancers. Both Th1 and Th2 CD4^+^ cell immunity to HPV is directed to HPV type 16 (HPV16) proteins E2, E6, E7 and L1 and CD8^+^ cell immunity is directed to E6. A peptide vaccine was developed to avoid the high costs associated with the production of DCs. Since proteins are inefficient at CD8^+^ cytotoxic T cell induction and short peptides do not produce adequate CD8 memory response due to lack of CD4^+^ helper cell induction, long peptides which are processed and presented by DCs were used in the vaccine. The clinical grade HPV16 SLP vaccine consists of 13 long peptides covering the entire length of the HPV16 E6 and E7 proteins. A phase I clinical trial involving patients with advanced cervical cancer found that the vaccine was safe and induced T cell IFN-γ responses [39,40]. A phase II trial of 20 patients with VIN2 pre-malignant lesions resulted in objective responses in >50% of patients after 12 months and 9 of the patients had complete and durable regression after 24 months. The strength of vaccine-induced HPV-16 T cell specific responses correlated with significant clinical response [41,42]. The vaccine is currently being tested in combination with chemotherapy.

Data from Pawel Kalinski, Hideho Okada and colleagues (University of Pittsburgh, Pittsburgh, PA, USA) showed that DCs maturing in different conditions induce different forms of immune responses. Data from Kalinski lab demonstrate that IL-12 produced by DC vaccines is an important factor in the induction of GrB+ effector CD8+ T cells (CTLs) expressing high levels of peripheral-homing receptors, CCR5 and CXCR3 [43]. A recently completed phase I/II clinical trial [44], demonstrated that the level of IL-12 production by DC vaccines is a predictive factor of delayed time to progression in patients with high grade recurrent gliomas (GBM+AA) treated with DC vaccines and poly-ICLC. Disease stabilization of 12 months or longer was observed in 9 of 22 accrued patients. Preclinical data, showing strong synergy between TLR3 ligands, IFNα, and COX2 inhibitors in the induction of the chemokine ligands for CCR5 and CXCR3 in tumor tissues [45], led to recent implementation of additional clinical studies testing the combined use of DC vaccines and tumor-conditioning factors in colorectal cancer.

## Oncolytic viruses

Vaccinia viruses infect and lyse some tumors. Recent clinical trials have found these oncolytic viruses to be safe and in some cases they have demonstrated tumor selectivity. Vaccinia infects most mammalian cell types and causes minor illness in humans. Boris Minev and collaborators (Genelux Corporation, San Diego, CA, USA and the University of California San Diego, La Jolla, CA, USA) have investigated the interactions of oncolytic viruses with the host immune system. Analysis of the mononuclear cell subset tropism of several oncolytic vaccinia virus constructs found that they preferentially infected monocytes and activated T cells, but were much less likely to infect B cells, NK cells and resting T cells. However, the viral amplification and cytotoxicity were greater in control cancer cells than in the mononuclear cells. Oncolytic vaccinia is now being used to treat patients with chronic myelomonocytic leukemia (CMML) in an early phase clinical trial.

Vaccinia has been engineered to express GM-CSF in order to modulate the tumor microenvironment and enhance anti-tumor immunity and has been used as an anti-tumor vaccine [46,47]. Another vaccinia-based therapy being tested in clinical trials is Fowlpox-GM-CSF TRICOM. Edmund Lattime and colleagues (Cancer Center of New Jersey, New Brunswick, NJ, USA) are testing fowlpox virus engineered to express GM-CSF plus TRICOM, a triad of costimulatory molecules, consisting of LFA-3, ICAM and B7.1. TRICOM provides T cell costimulation in the context of tumor antigen presentation. The recombinant fowlpox vector is being tested in patients with bladder cancer in a phase I clinical trial. PanVac is another vaccinia virus vaccine expressing TRICOM and two pancreatic cancer antigens, carcinoembryonic antigen (CEA) and mucin 1 (MUC-1). PanVac is being given intratumorally to patients with pancreatic cancer. PanVac has been given to 11 patients with locally advanced disease and although 10 patients in the trial remained metastasis-free, they all died of local disease progression.

Sung Yong Lee and colleagues (Johns Hopkins Medical Institutions, Baltimore, MD, USA) are investigating the use of vaccinia virus containing a vector expressing the chimeric protein calreticulin (CRT) linked to the human papilloma virus protein E7 (HPV-E7) (CRT/E7-VV) as a treatment for cervical cancer. E7 is a tumor antigen and CRT improves antigen presentation by MHC class I molecules and amplifies T cell responses. This virus is being tested for the treatment of cervical cancer using a murine model. The vaccine is given with cisplatin chemotherapy in order to change the tumor microenvironment by causing tumor lysis which increases antigen uptake by DCs. The authors reported that the combination of the vaccine and chemotherapy induced systemic anti-tumor effects and increase the number of E7 specific CD8+ T cells in the blood.

Vesicular stomatitis virus (VSV) is also being used in cancer immune therapy [48]. As shown by Richard Vile (Mayo Clinic, Rochester, MN, USA), VSV engineered to express interferon-β is being tested in a phase I clinical trial in patients with hepatocellular carcinoma. The VSV was administered as an intratumoral injection. Studies of tumor bearing mice have found that the mechanism by which VSV induces tumor lysis involves the induction of strong innate immune response when injected into tumors which likely is responsible for killing both infected and non-infected tumor cells. VSV is also being used to encode tumor associated antigens in order to prime strong anti-tumor T cell responses. In addition, melanoma cDNA libraries expressed in VSV can be used to identify tumor-associated antigens [49].

## Bacteria as cancer vaccines

Bacteria are also being used for cancer vaccines. Listeria monocytogenes has been engineered to express antigens and used to treat cancers [50]. Listeria preferentially infects monocytes and generates a strong innate immune response. ADXS11-001 (ADXS-HPV) LM-LLO is a live attenuated Listeria monocytogenes engineered to secrete a HPV-16-E7 fusion protein. This immunotherapy is designed to target HPV transformed cells and is being evaluated by Robert Petit (Advaxis, Inc, Princeton, NJ, USA) and Partha Basu (Chittaranjan National Cancer Institute, Kolkata, India) for the treatment of cervical cancer. Animal models have shown that ADXS-HPV increases the level of circulating T cells and reduces the levels of T_REG_ cells and MDSCs breaking immune tolerance. ADXS11-001 has been used in a phase II clinical trial of 110 patients with recurrent and refractory cervical cancer that had been treated with chemotherapy or radiotherapy. The patients received ADXS11-001 alone or ADXS11-001 plus cisplatin chemotherapy. Some patients experienced complete responses and partial responses, but no improvement in responses were noted in patients that received cisplatin chemotherapy in addition to ADXS11-001.

## Combining immunotherapy and targeted therapy for melanoma

Antoni Ribas (University of California Los Angeles, CA, USA) reported that traditional cancer chemotherapy or novel targeted therapies are being tested in combination to improve the effects of tumor immunotherapies. Several agents have been used to overcome these problems including a proteasome inhibitor (bortezomib) which sensitizes NK cells, a Bcl-2 inhibitor (ABT-737) which sensitizes T cells by making cancer cells sensitive to granzyme B induced apoptosis [51] and the HDAC inhibitor (LAQ824) which improves antigen presentation by tumor cells and enhances the function of T cells [52]. Another agent which sensitizes T cells has become available; the BRAF inhibitor vemurafenib or PLX4032. Initial studies of vemurafenib have been promising. Clinically, vemurafenib has been found to increase the quantity of T cell infiltrates in regressing melanomas. Studies of pmel-1 murine melanoma model have found that when given with ACT, vemurafenib increases immune cell function and possibly modulates the tumor microenvironment. Specifically vemurafenib paradoxically increased MAPK signaling, in vivo cytotoxic activity, and intratumoral cytokine secretion by adoptively transferred cells [53].

Many cancers have RAS mitogen-activated protein kinase (MAPK) oncogene mutations that lead to tumor immune evasion in part due to immune escape. One such mutation is the prevalent BRAF V600 mutation in melanoma. Recently, it has been discovered that the inhibition of BRAF increases the activity of immunotherapies. Antoni Ribas and colleagues demonstrated that in mouse models, BRAF inhibitors increase the in vivo cytotoxic activity and intratumoral cytokine secretion by adoptively transferred cells, without altering their expansion, distribution, or tumor accumulation. Moreover, a BRAF inhibitor, vemurafenib (PLX4032), is effective in treating patients with advanced melanoma [54].

The tyrosine kinase inhibitor imatinib was originally used to treat chronic myelogenous leukemia and Robert de Mateo and colleagues (Memorial Sloan Kettering Cancer Center, New York, NY, USA) have shown imatinib to have antitumor activity in the treatment of gastrointestinal stromal cell tumors (GIST) with mutations in KIT, BRAF and PDGFRα [55]. A clinical trial involving 694 patients with metastatic GIST found that imatinib resulted in partial responses or stable disease in about 80% of patients and median survival was almost 5 years [56]. In a second clinical trial, following surgical resection of GIST, 713 patients were randomized to receive either imatinib or no additional therapy. Disease recurrence and progression was delayed in patients treated with Imatinib, but it did not result in improved long-term disease free survival. Robert de Mateo and colleagues have also reported that the quantity of T cells in tumors post-imatinib therapy increased and the CD8/T_REG_ ratio improved in tumor samples from these studies. In addition, tumor associated macrophages (TAM) are likely involved with the responses of GIST to imatinib. TAM in GIST are predominantly inflammatory (M1), which is atypical. The number of TAM decreased in patients sensitive to imatinib but develop a type 2 phenotype post treatment. In contrast, the number of TAM increase and retain a type M1 phenotype in patients resistant to imatinib [57].

Laurence Zitvogel (Institute Gustave Roussy, Paris, France) reported that imatinib induces NK-mediated tumor control in mice by acting on c-kit in DCs to promote NK and DC cell cross talk [58]. The number of NK cells and T cells in GIST is greater than in other sarcomas. These NK cells and T cells are activated and produce Th1 cytokines. Imatinib treatment is associated with increased infiltration of GIST with NK cells and it also increases the secretion of IFN-γ by NK cells in blood and tumors. The infiltration of GIST by NK cells is predictive of response to imatinib as is the activation of circulating NK cells [59]. The molecular characteristics of NK cells are important in the response of GIST to imatinib. NK cells with different NKp30 alternate splicing isoforms vary in their production of IFN-γ and IL-12 and GIST patients with the NKp30c isoform don’t benefit from imatinib [60]. Serum biomarkers of imatinib responsiveness have been investigated and a target of NKp30, soluble B7-H6, has been found to be effective when used in combination with soluble MICA/B. In these studies, increased levels of sB7-H6 and decreased levels of sMIC were associated with poor prognosis.

Cetuximab is an antibody specific for epidermal growth factor receptor (EGFR), which when used in combination with chemotherapy and radiotherapy induces clinical responses in 15% to 20% of patients with head and neck cancer (HNC). Hyun-Bae Jie and colleagues (University of Pittsburgh Cancer Institute, Pittsburgh, PA, USA) found that a possible explanation of the limited response to this therapy may be related to FOXP3^+^ T_REG_ cells. The numbers of circulating and intratumoral FOXP3^+^ T_REG_ cells are increased in patients with HNC tumors. After cetuximab treatment their numbers increased further. Furthermore, patients with greater FOXP3+ T_REG_ cell levels were less likely to respond to cetuximab treatment.

## Radiotherapy to enhance immunotherapy

Radiation therapy is reported to be able to convert the tumor into an *in situ* vaccine by inducing tumor cell death and a pro-inflammatory microenvironment. The fact that this is a rare event suggests that radiotherapy usually has an immunosuppressive effect. Studies by Claire Vanpouille-Box and colleagues (NYU School of Medicine, New York, NY, USA) have found that radiotherapy causes immune suppression mediated by transforming growth factor β (TGFβ). They found that tumor-bearing mice treated with irradiation and a TGFβ neutralizing antibody have fewer lung metastases than mice treated with irradiation alone.

## Single cell high throughput technologies

An important aspect of cancer immunotherapy is measuring the immune response. Several advances have been made in high throughput single cell analysis. As shown by Alessandra Cesano (Nodality, South San Francisco, CA, USA) a new approach to evaluate immune cells known as the Single Cell Network Profile (SCNP) has been developed. SCNP is a multiparametric flow cytometry-based analysis that simultaneously measures, at the single-cell level, both extracellular surface markers and changes in intracellular signaling proteins in response to extracellular modulators. This approach allows for simultaneous functional measurements from multiple cell subpopulations without the need for prior cell separation [61,62].

Another approach to evaluate immune cells is single cell mass cytometry which is a multiparametric approach that combines single cell analysis with mass spectrometry. As discussed by Gary Nolan (Stanford School of Medicine, Stanford, CA, USA), cells are first labeled with antibodies conjugated with elemental isotopes, nebulized to free ions with high temperature (7500K) and then analyzed with mass cytometer. This approach allows the analysis of over 45 different parameters at the single cell level. Analysis of cells from cancer patients could be used to find common meta-clusters, surface markers or stem markers that can be different within and across patients (pre-post treatment or recurrent forms of the same cancer) [63,64].

Janet Siebert and collaborators (CytoAnalytics, Denver, CO, USA and the Robert W. Franz Cancer Center, Providence Cancer Center, Portland, OR, USA) have used another high throughput approach to identify biomarkers associated with longevity in melanoma patients treated with the anti-CTLA-4 antibody ipilimumab. The patients’ peripheral blood mononuclear cells were tested before and after treatment with a 12-parameter flow cytometry staining panel to delineate memory and effector T cell subsets and a 10-parameter panel to delineate T_REG_ cell subsets. The memory/effector cell panel identified 32 phenotypes associated with “long-lived” patients all of which were CD4^+^ cell phenotypes. In addition, the “long-lived” patients were more likely to have a large post therapy increase in early activated memory CD4^+^ T cells.

## Clinical immunotherapy guidelines for the treatment of melanoma

Howard Kaufman (Rush University, Chicago, IL, USA) reported that SITC has been developing Clinical Immunotherapy Guidelines (CIG) and the first guidelines are directed toward the treatment of melanoma. These guidelines have been drafted and have been posted on SITC web site and are open for comment. Future guidelines will address immunotherapy of genital-urinary tract malignancies and hematological malignancies.

## FDA update

Raj K. Puri, [US Food and Drug Administration (FDA), Center for Biologics Evaluation and Research (CBER), Bethesda, MD, USA], indicated that the FDA has recently posted several new guidelines on their website that are of interest to the SITC community. The new guidelines include: Guidance for the Industry: Clinical Considerations for Therapeutic Cancer Vaccines; Draft In Vitro Companion Diagnostic Guidance; and Draft Guidance for Industry: Codevelopment of two or more Unmarketed Investigational Drugs for Use in Combination. The FDA Office of Cellular, Tissue and Gene Therapies (OCTGT) also has some useful tools available on the FDA website including the OCTGT Learning Webinar series and References for the Regulatory Process of the Office of Cellular, Tissue and Gene Therapies (OCTGT). The FDA also has a new office of Hematology and Oncology Products.

## Conclusions

Immunotherapy of cancer continues to grow. The success of TIL therapy is being documented at several centers and TIL production is becoming simpler and less expensive. Anti-CD19 CAR T cell therapy continues to show promising preliminary results and its use is growing while new CAR therapies are being developed. T cells used in ACT are being engineered to express high affinity TCRs and IL-12. Methods to produce T cells with stem cell characteristics are being developed for use in ACT in order to improve the survival and proliferation adoptively transferred T cells. Several DC therapies have proven to reliably induce peripheral blood T cell responses and T cell and B cell infiltration into the tumor microenvironment. Additional antibodies capable of PD-1/PD-L1 and CTLA-4 pathway blockade are being developed. Initial studies of immunotherapy combinations with targeted therapies have been promising, as have vaccines making use of oncolytic viruses, Listeria monocytogenes and mRNA.

## Competing interests

DHM has intellectual property interests in the therapeutic use of IDO and IDO inhibitors, and receives consulting income and research support from NewLink Genetics, Inc. AAS is a salaried employee of Genelux Corporation and has a personal financial interest in Genelux Corporation.

## Authors’ contributions

DFS, CJJM and AR organized the meeting. AC, AR, MAC, BC-A, TFG, PDG, PK, HLK, SNK, FM, WM, CJJM, DHM, DJP, NPR, SAR, RKP, HS, DFS, AAS, CY and LZ organized meeting sessions. DFS, LC and SC drafted the manuscript. All authors read and approved the final manuscript.

## References

[B1] Schreiber RD Old LJ Smyth MJ Cancer immunoediting: integrating immunity’s roles in cancer suppression and promotion Science 2011 1 1565 1570 21436444 10.1126/science.1203486

[B2] Szajnik M Czystowska M Szczepanski MJ Mandapathil M Whiteside TL Tumor-derived microvesicles induce, expand and up-regulate biological activities of human regulatory T cells (Treg) PLoS One 2010 1 e11469 20661468 10.1371/journal.pone.0011469 PMC2908536

[B3] Johnson TS Munn DH Host indoleamine 2,3-dioxygenase: contribution to systemic acquired tumor tolerance Immunol Invest 2012 1 765 797 23017145 10.3109/08820139.2012.689405

[B4] Munn DH Sharma MD Hou D Baban B Lee JR Antonia SJ Expression of indoleamine 2,3-dioxygenase by plasmacytoid dendritic cells in tumor-draining lymph nodes J Clin Invest 2004 1 280 290 15254595 10.1172/JCI21583 PMC449750

[B5] Blank C Brown I Peterson AC Spiotto M Iwai Y Honjo T PD-L1/B7H-1 inhibits the effector phase of tumor rejection by T cell receptor (TCR) transgenic CD8+ T cells Cancer Res 2004 1 1140 1145 14871849 10.1158/0008-5472.can-03-3259

[B6] Westwood JA Darcy PK Guru PM Sharkey J Pegram HJ Amos SM Three agonist antibodies in combination with high-dose IL-2 eradicate orthotopic kidney cancer in mice J Transl Med 2010 1 42 20426873 10.1186/1479-5876-8-42 PMC2873376

[B7] Messina JL Fenstermacher DA Eschrich S Qu X Berglund AE Lloyd MC 12-Chemokine gene signature identifies lymph node-like structures in melanoma: potential for patient selection for immunotherapy? Sci Rep 2012 1 765 23097687 10.1038/srep00765 PMC3479449

[B8] Klebanoff CA Finkelstein SE Surman DR Lichtman MK Gattinoni L Theoret MR IL-15 enhances the in vivo antitumor activity of tumor-reactive CD8+ T cells Proc Natl Acad Sci U S A 2004 1 1969 1974 14762166 10.1073/pnas.0307298101 PMC357036

[B9] Bergamaschi C Bear J Rosati M Beach RK Alicea C Sowder R Circulating IL-15 exists as heterodimeric complex with soluble IL-15Ralpha in human and mouse serum Blood 2012 1 e1 e8 22496150 10.1182/blood-2011-10-384362 PMC3390963

[B10] Stroncek DF Berger C Cheever MA Childs RW Dudley ME Flynn P New directions in cellular therapy of cancer: a summary of the summit on cellular therapy for cancer J Transl Med 2012 1 48 22420641 10.1186/1479-5876-10-48 PMC3362772

[B11] Rosenberg SA Yang JC Sherry RM Kammula US Hughes MS Phan GQ Durable complete responses in heavily pretreted patients with metastatic melanoma using T cell transfer immunotherapy Clin Cancer Res 2011 1 4550 4557 21498393 10.1158/1078-0432.CCR-11-0116 PMC3131487

[B12] Chapuis AG Thompson JA Margolin KA Rodmyre R Lai IP Dowdy K Transferred melanoma-specific CD8+ T cells persist, mediate tumor regression, and acquire central memory phenotype Proc Natl Acad Sci U S A 2012 1 4592 4597 22393002 10.1073/pnas.1113748109 PMC3311364

[B13] Dudley ME Gross CA Langhan MM Garcia MR Sherry RM Yang JC CD8+ enriched “young” tumor infiltrating lymphocytes can mediate regression of metastatic melanoma Clin Cancer Res 2010 1 6122 6131 20668005 10.1158/1078-0432.CCR-10-1297 PMC2978753

[B14] Jin J Sabatino M Somerville R Wilson JR Dudley ME Stroncek DF Simplified method of the growth of human tumor infiltrating lymphocytes in gas-permeable flasks to numbers needed for patient treatment J Immunother 2012 1 283 292 22421946 10.1097/CJI.0b013e31824e801f PMC3315105

[B15] Ye Q Loisiou M Levine BL Suhoski MM Riley JL June CH Engineered artificial antigen presenting cells facilitate direct and efficient expansion of tumor infiltrating lymphocytes J Transl Med 2011 1 131 21827675 10.1186/1479-5876-9-131 PMC3162913

[B16] Wang A Chandran S Shah SA Chiu Y Paria BC Aghamolla T The stoichiometric production of IL-2 and IFN-gamma mRNA defines memory T cells that can self-renew after adoptive transfer in humans Sci Transl Med 2012 1 149ra120 10.1126/scitranslmed.3004306 PMC6453124 22932225

[B17] Radvanyi LG Bernatchez C Zhang M Fox P Miller P Chacon J Specific lymphocyte subsets predict response to adoptive cell therapy using expanded autologous tumor-infiltrating lymphocytes in metastatic melanoma patients Clin Cancer Res 2012 1 6758 6770 23032743 10.1158/1078-0432.CCR-12-1177 PMC3525747

[B18] Yao X Ahmadzadeh M Lu YC Liewehr DJ Dudley ME Liu F Levels of peripheral CD4(+)FoxP3(+) regulatory T cells are negatively associated with clinical response to adoptive immunotherapy of human cancer Blood 2012 1 5688 5696 22555974 10.1182/blood-2011-10-386482 PMC3382928

[B19] Li Y Yee C IL-21 mediated Foxp3 suppression leads to enhanced generation of antigen-specific CD8+ cytotoxic T lymphocytes Blood 2008 1 229 235 17921346 10.1182/blood-2007-05-089375 PMC2200808

[B20] Li Y Bleakley M Yee C IL-21 influences the frequency, phenotype, and affinity of the antigen-specific CD8 T cell response J Immunol 2005 1 2261 2269 16081794 10.4049/jimmunol.175.4.2261

[B21] Lang PA Lang KS Xu HC Grusdat M Parish IA Recher M Natural killer cell activation enhances immune pathology and promotes chronic infection by limiting CD8+ T-cell immunity Proc Natl Acad Sci U S A 2012 1 1210 1215 22167808 10.1073/pnas.1118834109 PMC3268324

[B22] Zhang L Feldman SA Zheng Z Chinnasamy N Xu H Nahvi AV Evaluation of gamma-retroviral vectors that mediate the inducible expression of IL-12 for clinical application J Immunother 2012 1 430 439 22576348 10.1097/CJI.0b013e31825898e8 PMC3358728

[B23] Kerkar SP Goldszmid RS Muranski P Chinnasamy D Yu Z Reger RN IL-12 triggers a programmatic change in dysfunctional myeloid-derived cells within mouse tumors J Clin Invest 2011 1 4746 4757 22056381 10.1172/JCI58814 PMC3226001

[B24] Kerkar SP Muranski P Kaiser A Boni A Sanchez-Perez L Yu Z Tumor-specific CD8+ T cells expressing interleukin-12 eradicate established cancers in lymphodepleted hosts Cancer Res 2010 1 6725 6734 20647327 10.1158/0008-5472.CAN-10-0735 PMC2935308

[B25] Robbins PF Morgan RA Feldman SA Yang JC Sherry RM Dudley ME Tumor regression in patients with metastatic synovial cell sarcoma and melanoma using genetically engineered lymphocytes reactive with NY-ESO-1 J Clin Oncol 2011 1 917 924 21282551 10.1200/JCO.2010.32.2537 PMC3068063

[B26] Morgan RA Dudley ME Wunderlich JR Hughes MS Yang JC Sherry RM Cancer regression in patients after transfer of genetically engineered lymphocytes Science 2006 1 126 129 16946036 10.1126/science.1129003 PMC2267026

[B27] Kochenderfer JN Dudley ME Feldman SA Wilson WH Spaner DE Maric I B-cell depletion and remissions of malignancy along with cytokine-associated toxicity in a clinical trial of anti-CD19 chimeric-antigen-receptor-transduced T cells Blood 2012 1 2709 2720 22160384 10.1182/blood-2011-10-384388 PMC3327450

[B28] Porter DL Levine BL Kalos M Bagg A June CH Chimeric antigen receptor-modified T cells in chronic lymphoid leukemia N Engl J Med 2011 1 725 733 21830940 10.1056/NEJMoa1103849 PMC3387277

[B29] Brentjens RJ Riviere I Park JH Davila ML Wang X Stefanski J Safety and persistence of adoptively transferred autologous CD19-targeted T cells in patients with relapsed or chemotherapy refractory B-cell leukemias Blood 2011 1 4817 4828 21849486 10.1182/blood-2011-04-348540 PMC3208293

[B30] Kloss CC Condomines M Cartellieri M Bachmann M Sadelain M Combinatorial antigen recognition with balanced signaling promotes selective tumor eradication by engineered T cells Nat Biotechnol 2012 1 71 75 23242161 10.1038/nbt.2459 PMC5505184

[B31] Hanada K Restifo NP Double or nothing on cancer immunotherapy Nat Biotechnol 2013 1 33 34 23302931 10.1038/nbt.2471 PMC6352981

[B32] Gattinoni L Klebanoff CA Restifo NP Paths to stemness: building the ultimate antitumour T cell Nat Rev Cancer 2012 1 671 684 22996603 10.1038/nrc3322 PMC6352980

[B33] Restifo NP Dudley ME Rosenberg SA Adoptive immunotherapy for cancer: harnessing the T cell response Nat Rev Immunol 2012 1 269 281 22437939 10.1038/nri3191 PMC6292222

[B34] Klebanoff CA Gattinoni L Restifo NP Sorting through subsets: which T-cell populations mediate highly effective adoptive immunotherapy? J Immunother 2012 1 651 660 23090074 10.1097/CJI.0b013e31827806e6 PMC3501135

[B35] Hinrichs CS Spolski R Paulos CM Gattinoni L Kerstann KW Palmer DC IL-2 and IL-21 confer opposing differentiation programs to CD8+ T cells for adoptive immunotherapy Blood 2008 1 5326 5333 18276844 10.1182/blood-2007-09-113050 PMC2396726

[B36] Singh H Figliola MJ Dawson MJ Huls H Olivares S Switzer K Reprogramming CD19-specific T cells with IL-21 signaling can improve adoptive immunotherapy of B-lineage malignancies Cancer Res 2011 1 3516 3527 21558388 10.1158/0008-5472.CAN-10-3843 PMC3096697

[B37] Wolfl M Merker K Morbach H Van Gool SW Eyrich M Greenberg PD Primed tumor-reactive multifunctional CD62L+ human CD8+ T cells for immunotherapy Cancer Immunol Immunother 2011 1 173 186 20972785 10.1007/s00262-010-0928-8 PMC3043230

[B38] Sharma A Koldovsky U Xu S Mick R Roses R Fitzpatrick E HER-2 pulsed dendritic cell vaccine can eliminate HER-2 expression and impact ductal carcinoma in situ Cancer 2012 1 4354 4362 22252842 10.1002/cncr.26734 PMC3330145

[B39] Kenter GG Welters MJ Valentijn AR Lowik MJ der Berends-van Meer DM Vloon AP Phase I immunotherapeutic trial with long peptides spanning the E6 and E7 sequences of high-risk human papillomavirus 16 in end-stage cervical cancer patients shows low toxicity and robust immunogenicity Clin Cancer Res 2008 1 169 177 18172268 10.1158/1078-0432.CCR-07-1881

[B40] Welters MJ Kenter GG Piersma SJ Vloon AP Lowik MJ Der Berends-van Meer DM Induction of tumor-specific CD4+ and CD8+ T-cell immunity in cervical cancer patients by a human papillomavirus type 16 E6 and E7 long peptides vaccine Clin Cancer Res 2008 1 178 187 18172269 10.1158/1078-0432.CCR-07-1880

[B41] Kenter GG Welters MJ Valentijn AR Lowik MJ Der Berends-van Meer DM Vloon AP Vaccination against HPV-16 oncoproteins for vulvar intraepithelial neoplasia N Engl J Med 2009 1 1838 1847 19890126 10.1056/NEJMoa0810097

[B42] Melief CJ Treatment of established lesions caused by high-risk human papilloma virus using a synthetic vaccine J Immunother 2012 1 215 216 22421938 10.1097/CJI.0b013e318248f17f

[B43] Watchmaker PB Berk E Muthuswamy R Mailliard RB Urban JA Kirkwood JM Independent regulation of chemokine responsiveness and cytolytic function versus CD8+ T cell expansion by dendritic cells J Immunol 2010 1 591 597 20018619 10.4049/jimmunol.0902062 PMC2922038

[B44] Okada H Kalinski P Ueda R Hoji A Kohanbash G Donegan TE Induction of CD8+ T-cell responses against novel glioma-associated antigen peptides and clinical activity by vaccinations with {alpha}-type 1 polarized dendritic cells and polyinosinic-polycytidylic acid stabilized by lysine and carboxymethylcellulose in patients with recurrent malignant glioma J Clin Oncol 2011 1 330 336 21149657 10.1200/JCO.2010.30.7744 PMC3056467

[B45] Muthuswamy R Berk E Junecko BF Zeh HJ Zureikat AH Normolle D NF-kappaB hyperactivation in tumor tissues allows tumor-selective reprogramming of the chemokine microenvironment to enhance the recruitment of cytolytic T effector cells Cancer Res 2012 1 3735 3743 22593190 10.1158/0008-5472.CAN-11-4136 PMC3780565

[B46] Mastrangelo MJ Maguire HC Jr Eisenlohr LC Laughlin CE Monken CE McCue PA Intratumoral recombinant GM-CSF-encoding virus as gene therapy in patients with cutaneous melanoma Cancer Gene Ther 1999 1 409 422 10505851 10.1038/sj.cgt.7700066

[B47] Gomella LG Mastrangelo MJ McCue PA Maguire HC Jr Mulholland SG Lattime EC Phase i study of intravesical vaccinia virus as a vector for gene therapy of bladder cancer J Urol 2001 1 1291 1295 11547060

[B48] Rommelfanger DM Wongthida P Diaz RM Kaluza KM Thompson JM Kottke TJ Systemic combination virotherapy for melanoma with tumor antigen-expressing vesicular stomatitis virus and adoptive T-cell transfer Cancer Res 2012 1 4753 4764 22836753 10.1158/0008-5472.CAN-12-0600 PMC3893932

[B49] Pulido J Kottke T Thompson J Galivo F Wongthida P Diaz RM Using virally expressed melanoma cDNA libraries to identify tumor-associated antigens that cure melanoma Nat Biotechnol 2012 1 337 343 22426030 10.1038/nbt.2157 PMC3891505

[B50] Wallecha A French C Petit R Singh R Amin A Rothman J Lm-LLO-based immunotherapies and HPV-associated disease J Oncol 2012 1 2 542851 22481930 10.1155/2012/542851 PMC3307007

[B51] Sutton VR Sedelies K Dewson G Christensen ME Bird PI Johnstone RW Granzyme B triggers a prolonged pressure to die in Bcl-2 overexpressing cells, defining a window of opportunity for effective treatment with ABT-737 Cell Death Dis 2012 1 e344 22764103 10.1038/cddis.2012.73 PMC3406577

[B52] Vo DD Prins RM Begley JL Donahue TR Morris LF Bruhn KW Enhanced antitumor activity induced by adoptive T-cell transfer and adjunctive use of the histone deacetylase inhibitor LAQ824 Cancer Res 2009 1 8693 8699 19861533 10.1158/0008-5472.CAN-09-1456 PMC2779578

[B53] Koya RC Mok S Otte N Blacketor KJ Comin-Anduix B Tumeh PC BRAF inhibitor vemurafenib improves the antitumor activity of adoptive cell immunotherapy Cancer Res 2012 1 3928 3937 22693252 10.1158/0008-5472.CAN-11-2837 PMC3422880

[B54] Sosman JA Kim KB Schuchter L Gonzalez R Pavlick AC Weber JS Survival in BRAF V600-mutant advanced melanoma treated with vemurafenib N Engl J Med 2012 1 707 714 22356324 10.1056/NEJMoa1112302 PMC3724515

[B55] Dematteo RP Personalized therapy: prognostic factors in gastrointestinal stromal tumor (GIST) J Gastrointest Surg 2012 1 1645 1647 22752549 10.1007/s11605-012-1944-0 PMC3707794

[B56] Blanke CD Demetri GD von Mehren M Heinrich MC Eisenberg B Fletcher JA Long-term results from a randomized phase II trial of standard- versus higher-dose imatinib mesylate for patients with unresectable or metastatic gastrointestinal stromal tumors expressing KIT J Clin Oncol 2008 1 620 625 18235121 10.1200/JCO.2007.13.4403

[B57] Dematteo RP Ballman KV Antonescu CR Maki RG Pisters PW Demetri GD Adjuvant imatinib mesylate after resection of localised, primary gastrointestinal stromal tumour: a randomised, double-blind, placebo-controlled trial Lancet 2009 1 1097 1104 19303137 10.1016/S0140-6736(09)60500-6 PMC2915459

[B58] Borg C Terme M Taieb J Menard C Flament C Robert C Novel mode of action of c-kit tyrosine kinase inhibitors leading to NK cell-dependent antitumor effects J Clin Invest 2004 1 379 388 15286804 10.1172/JCI21102 PMC489961

[B59] Menard C Blay JY Borg C Michiels S Ghiringhelli F Robert C Natural killer cell IFN-gamma levels predict long-term survival with imatinib mesylate therapy in gastrointestinal stromal tumor-bearing patients Cancer Res 2009 1 3563 3569 19351841 10.1158/0008-5472.CAN-08-3807

[B60] Delahaye NF Rusakiewicz S Martins I Menard C Roux S Lyonnet L Alternatively spliced NKp30 isoforms affect the prognosis of gastrointestinal stromal tumors Nat Med 2011 1 700 707 21552268 10.1038/nm.2366

[B61] Cesano A Parkinson D Applications of multiparametric flow cytometry: providing new insights into biology to bridge the gap between research discovery and clinical application Cytometry A 2012 1 732 733 22730060 10.1002/cyto.a.22087

[B62] Longo DM Louie B Putta S Evensen E Ptacek J Cordeiro J Single-cell network profiling of peripheral blood mononuclear cells from healthy donors reveals age- and race-associated differences in immune signaling pathway activation J Immunol 2012 1 1717 1725 22246624 10.4049/jimmunol.1102514 PMC3517183

[B63] Bendall SC Simonds EF Qiu P Amir e Krutzik PO Finck R Single-cell mass cytometry of differential immune and drug responses across a human hematopoietic continuum Science 2011 1 687 696 21551058 10.1126/science.1198704 PMC3273988

[B64] Bodenmiller B Zunder ER Finck R Chen TJ Savig ES Bruggner RV Multiplexed mass cytometry profiling of cellular states perturbed by small-molecule regulators Nat Biotechnol 2012 1 858 867 22902532 10.1038/nbt.2317 PMC3627543

